# Commercial video games and cognitive functions: video game genres and modulating factors of cognitive enhancement

**DOI:** 10.1186/s12993-020-0165-z

**Published:** 2020-02-03

**Authors:** Eunhye Choi, Suk-Ho Shin, Jeh-Kwang Ryu, Kyu-In Jung, Shin-Young Kim, Min-Hyeon Park

**Affiliations:** 1 Department of Psychiatry, Eunpyeong St. Mary’s Hospital, College of Medicine, The Catholic University of Korea, 1021 Tongil-ro, Eunpyeong-gu, Seoul, Republic of Korea; 2 Dr. Shin’s Child and Adolescent Psychiatry Clinic, Seoul, Republic of Korea; 3 Institute for Cognitive Science, Seoul National University, Seoul, Republic of Korea

**Keywords:** Cognitive functions, Commercial video games, Genres of video games, Modulating factors

## Abstract

**Background:**

Unlike the emphasis on negative results of video games such as the impulsive engagement in video games, cognitive training studies in individuals with cognitive deficits showed that characteristics of video game elements were helpful to train cognitive functions. Thus, this study aimed to have a more balanced view toward the video game playing by reviewing genres of commercial video games and the association of video games with cognitive functions and modulating factors. Literatures were searched with search terms (e.g. genres of video games, cognitive training) on database and Google scholar.

**Results:**

video games, of which purpose is players’ entertainment, were found to be positively associated with cognitive functions (e.g. attention, problem solving skills) despite some discrepancy between studies. However, the enhancement of cognitive functions through video gaming was limited to the task or performance requiring the same cognitive functions. Moreover, as several factors (e.g. age, gender) were identified to modulate cognitive enhancement, the individual difference in the association between video game playing and cognitive function was found.

**Conclusion:**

Commercial video games are suggested to have the potential for cognitive function enhancement. As understanding the association between video gaming and cognitive function in a more balanced view is essential to evaluate the potential outcomes of commercial video games that more people reported to engage, this review contributes to provide more objective evidence for commercial video gaming.

## Background

Despite objective research findings which addressed both positive and negative sides of video game (VG) playing, the negativity of VG playing, such as the obsession with VG playing [1] and increased feeling and thoughts of aggression [2], has been more focused. The World Health Organization announced the inclusion of “gaming disorder” in the category of addictive behavior disorders in the 11th International Statistical Classification of Diseases and Related Health Problems [3]. However, violent VGs, which were reported to increase aggression [1], were found to be positively associated with visuo-spatial abilities without the influence on aggression [4]. It seemed because action video games (AVGs), which can include violent elements, do not always refer to violent VGs [5]. Consistent with the argument that VGs should be regarded as one type of learning [6], VGs were also found to enhance cognitive functions better than conventional methods of learning [7] by conveying information in a different way from traditional media [8]. Taken together, unlike the emphasis on the negativity on VG playing, VGs, which provide players with richer environment of cognitive, emotional and social experience, are suggested to enhance their cognitive functions [9] by simulating cognitive processes, which are activated in real world, in the process of completing VG tasks [10]. Thus, it is important to understand commercial VGs in a more balanced view. In order to deepen the understanding of commercial VGs, this study reviews genres of VGs, cognitive functions identified to be positively associated with VG playing, and factors for individual difference in the association between VGs and cognitive enhancement.

## Methods

Literatures were searched on Google Scholar and database (e.g. PubMed, PsychInfo) without date restriction. All designs of studies, found through the search (e.g. cross-sectional studies, training studies and review papers), were included. Search terms for the first section, reviewing genres of VGs, were “genres of (video) games”. “Serious games” was additionally used search term to make the distinction between serious games and commercial VGs. In the second section discussing the association between VG playing and cognitive functions, search terms were “video game (playing)”, “cognitive function/training” and “the association between VG and cognitive function”. Searched literatures for commercial VGs were categorized as six different cognitive functions. As AVGs were found to be highly investigated among various genres of VGs in the search, more literatures for AVGs were included in the second section. Based on literatures for the second section and additionally searched literatures with search terms (e.g. “age and video game”), the last section discussed the factors that were considered as variables for the individual difference in the association of VGs with cognitive functions.

## Genres of VGs

Cognitive trainings, having components of VGs (i.e. adapting the difficulty level based on the performance and instantly providing the feedback) [11], were found to be effective through the individualization of the training (see Table 1). Constant provision of feedback was helpful for self-monitoring of the progress in VGs [12] in that players were able to change their decisions based on the feedback [13]. VGs, which are suggested to have the potential to train cognitive functions, seem to be divided into two genres depending on the purpose of the development: serious games and commercial VGs. Serious games, which are developed for learning and changes of behavior in various areas such as business, education, healthcare and policies of the government [14, 15], were found to be more effective learning methods compared to conventional methods of learning when people played multiple sessions in groups with supplementary instructions [16]. Unlike serious games, commercial VGs were designed for the entertainment of players [17]. Although it is not designed for learning, commercial VGs provide players with goal-driven environment that they face various challenges and conflicts [18]. Players were found to execute their cognitive skills in a more integrated way by playing commercial VGs [19]. Moreover, people are more motivated to play commercial VGs [20]. Taken together, the potential influence of commercial VGs on the enhancement of cognitive functions is suggested. Thus, this section focuses on the classification of commercial VGs.

**Table 1:** Two cognitive training studies in individuals with cognitive deficits

Study	Participants	Training procedure	Results of the training
[21]	Individuals with different extent of TBI (N = 3)	Computer-based treatment training attention and executive function in order to enhance higher cognitive skills (e.g. problem solving skill)	The improvement in cognitive functions despite the individual difference in the extent to which the functions were improved
[11]	Children_SPD_ (N = 10), children_SPD+ADHD_(N = 17), TDC(N = 20)	Four-week-long training conducted by using laboratory-based games to improve cognitive functions/cognitive control and 9-month follow-up test	Improved cognitive control in all children but greater enhancement in children_SPD+ADHD_ showing more attentional deficits than other two groups of children; the persistent training effect found based on parental reports of children’s attentional skill

Notes. TBI stands for traumatic brain injuries (i.e. diverse cognitive deficits with greater extent of severity), SPD stands for sensory processing dysfunction (i.e. the exhibition of exaggerated behavioral response to sensory stimuli), ADHD stands for attention deficit/hyperactivity dysfunction (i.e. difficulty to sustain attention and/or the exhibition of hyperactive movements), TDC stands for typically developing children

Based on four literatures [17, 22–24], five genres of VGs were identified (see Table 2). Firstly identified genre of commercial VGs is traditional games (TGs) such as puzzle, card and board VGs [23]. Secondly identified genre is simulation games (SGs) (i.e. sports or driving VGs [22], Sims building up towns or communities [24]). Thirdly identified genre is strategy video games (SVGs) referring to VG that players generally play in the global view by focusing on visual information [22] and planning the strategies [17]. As Table 2 shows, SVGs are sub-divided into real-time strategy (RTS) and turn-based strategy (FBS) depending on the way mental process occurs. In SVGs (e.g. Starcraft), expert play (i.e. the integration and contextualization of VG-world activities) is highly associated with the best possible outcomes of VGs [22]. Fourthly identified genre is action video games (AVGs) that are characterized by the existence of a static physical locator connecting gaze and actions of players in the game environment [22]. As shown in Table 2, AVGs are divided into first-person shooters (FPS) and third-person games (TPG) depending on the perspective of the players in the game. The final genre identified in the literatures is fantasy games (FGs). They can be defined as VGs where players explore the game environment in relatively slow pace in order to solve problems [17] and that focus on the imagination by offering fantasy environment with rules to players [22]. Among described sub-genres of FGs in Table 2, role-playing games (RPGs) are the starting point where the notion of VG community was formed [22]. Massive Multi-player Online RPGs (MMORPGs) are VGs where social and participatory aspects are emphasized by providing the VG itself as the social arena [22].

**Table 2:** Five genres of VGs

Genres	Sub-genres	Explanation
TGs		VGs that have been played in other medium for relatively long time [23]
SGs		VGs that are played to do relatively daily activities [22] or that are played based on players’ imagination for things that are difficult to be realized in real world [24]
SVGs	RTS	VGs where players take an active role in making the desired results by engaging relevant thinking process in VG playing; thinking the strategies by themselves [22]
	TBS	VGs where VG guides the thinking process of players [22]
AVGs	FPS	VGs played in the perspective of players [22]
	TPGs	VGs played by using the avatars of the players whose experience relies on the arrangement of space and time in game environments [22]
FGs	RPGs	VGs where players take the active control over avatars in the exploration of the environment, completion of quests and competing/battling with enemies [17]
	Adventure games	VGs that players explore and investigate the VG environment in slow pace by focusing on problem solving [17]

Although commercial VGs are classified as five genres in this review, the categorization of VGs seems to vary depending on the criteria for the classification (e.g. interaction type that players experience in VG environments) [17]. That is, same VGs can be classified as different genres depending on the aspect the researcher focused. For example, RPGs, which were categorized as ‘FGs’ based on characteristics of the VG environment [17], were categorized as ‘SVGs’ based on the way players performed in VGs [25]. Thus, more standardized categorizations of VGs are required by conducting further studies in order to more accurately investigate the association between VG playing and cognitive improvement. However, despite this limitation found in the genre classification, it is expected that different genres would be associated with different cognitive functions. It is because players face different designs of VG environments and show the different way of playing. Thus, the association of different VGs with cognitive function is reviewed in the next section.

## Cognitive functions identified to be positively related to VG playing

Although cognitive functions are found to be trained through VG playing during relatively short period, enhanced type of cognitive functions depends on genres of VGs [20]. In this section, the association of different genres with cognitive functions is reviewed. The transfer effect of VGs (i.e. the extent to which cognitive improvement associated with VGs is transferred into untrained cognitive functions) is also discussed. Six cognitive functions are identified to be positively associated with VGs.

Firstly identified cognitive function is attention. Frequent VG players were better at sustaining attention [26], and players of working memory (WM) and reasoning casual VGs showed the improvement in divided attention [19]. Compared to other genres of VGs played with slow pace, AVGs were highly associated with improvement in selective attention [27] which refers to the allocation of attention to relevant information [28]. FPS players were found to efficiently allocate attention through the improvement in the top-down process of attention [29]. Although Leauge of Legends (LoL) top-ranking players were better at selective attention than players with lower level skills and less gaming experience, one hour of AVG session resulted in better selective attention in less skilled players [30]. Players of AVGs and adventure games also showed attenuated attentional blink [31], which refers to the failure to detect and process the target that was subsequently presented right after the previously processed target [32]. That is, the training of AVGs, but not other genres of VGs (e.g. TGs and SGs), improved the recovery from attentional blink [32]. Furthermore, the improvement in attention, found to be associated with VGs [33, 34], accompany changes in brain regions. While dorsal fronto-parietal network, which is involved in top-down process of attention [35], was more activated with increased attentional demands in non-VG players or players of other genres (e.g. SVGs), AVG players barely recruited this network and showed reduced activation in visual motion sensitive area (MT/MST) of which activation results from moving distracters [34]. Changes in brain activation suggested that AVG players were better at filtering information and efficiently allocating attention to important information. Moreover, AVG experience was found to be positively associated with the plasticity of white matter network in regions (e.g. prefrontal cortex; PFC) [36] that involves in cognitive control (i.e. goal-directed neural process) [37]. Even older players showed increased activation in right dorsolateral PFC (DLPFC) [38]. Taken together, VG playing, especially AVG playing, is associated with the enhancement of visual attention that takes an important role in the efficient processing of information [39].

Based on the interaction between visual attention and WM [40], secondly identified cognitive function is WM that refers to the maintenance of presented visual stimuli [41]. When the association between casual WM reasoning games and cognitive function enhancement was investigated, the enhancement in WM was not found [19]. However, frequent VG playing was associated with the improvement in WM capacity [26]. The 20 h of training to play hidden-object and memory matrix VGs resulted in the improvement in spatial WM [32]. Although 20 h of AVG training did not enhance spatial WM [32], 30 h of AVG training during 1 month, compared to the training of SGs, resulted in the enhancement in visual WM [28]. Extensive experience of AVG playing was associated with better visual WM capacity [42]. FPS players showed more accurate and faster processing of relevant information with better WM capacity compared to non-players [43]. That is, AVG players showed more precise and detailed visual representation [44] and performed better in a change detection task than non-VG players [45]. When AVGs were played in long term, salience network, involved in the detection of visual stimuli (e.g. anterior cingulated cortex and anterior insula) and central executive network, involved in attentional control and WM such as DLPFC and posterior parietal cortex, were highly integrated [46]. AVG players showed improved WM capacity by efficiently allocating attention to important information [42]. These findings suggested that playing VGs, especially AVGs, is suggested to have the potential to enhance WM that is important for the learning of skills and the acquisition of knowledge [41, 42]. However, as it is unclear whether the discrepancy between AVG training studies result from the different duration of trainings or different aspects of WM, further studies are required.

Thirdly identified cognitive function is visuo-spatial function referring to perception, recognition, and manipulation of visual stimuli (e.g. visuo-motor coordination, navigation skill) [27]. Enhanced spatial cognition was reported in players of Tetris [47], which can be classified as one of TGs, and playing TGs (i.e. logic/puzzle games) was associated with gray matter (GM) volume in bilateral entorhinal cortex [48] that is involved in navigation [49]. AVGs and SVGs were also found to be associated with the enhanced visuo-spatial function [50]. Ten hours of AVG training resulted in better navigation skills through the adoption of response strategy, which indirectly measure/indicate the volume of hippocampus and striatum [51]. Consistently, AVG players, who were trained to play SuperMario for 2 months, showed the improvement in the processing of spatial information and the coordination of visuo-motor function along with larger GM volume in brain regions (i.e. right hippocampus, right DLPFC and bilateral cerebellum) [52]. Moreover, increases in white matter connections between occipital and parietal areas were found in RTS players compared to non-video gamers [53]. Furthermore, adolescents with more experience of VG playing showed thicker cortex in left frontal eye-fields that engage in allocating visuo-spatial attention and integrating relevant visuo-motor information [54]. That is, playing VGs was found to be associated with neural plasticity in brain regions involved in navigation and visual attention (i.e. bilateral entorhinal cortex, hippocampal and occipital GM volume) [48]. Taken together, although the exact duration of VG training for the detection of structural changes in brain was not identified [54], VGs are suggested to be associated with the enhancement in visuo-spatial function.

Fourthly identified cognitive function is probabilistic learning that refers to the usage of declarative memory to resolve the uncertainty [55]. Fifty hours of AVG training in non-VG players increased the efficiency to use not only visually but also auditorily available information [56]. AVG players also showed higher activation in brain regions involved in visual imagery, semantic memory and cognitive control (e.g. hippocampus, precuneus, thalamus) compared to non-AVG players [55]. Higher activation in hippocampus in AVG players was related to more pronounced usage of declarative knowledge [55]. Moreover, the cortex of left DLPFC, involved in resolving the ambiguity by using the cues in the environments, was found to be thicker in adolescents reporting longer duration of VG playing, suggesting players became better at resolving the ambiguity efficiently through VG playing [54]. It can be concluded that VG playing could enhance the probabilistic learning through the efficient use of evidence presented in the environment of VG.

Fifthly identified cognitive function is problem solving skills. Problem solving skills were improved more through a puzzle VG compared to cognitive training game [57]. Adolescents, playing strategic VGs (i.e. SVGs, RPGs) more frequently during 4 years of high school period, also showed better skill to solve problems [25]. Playing strategic VGs, but not fast-paced VGs, was also found to be associated with better academic achievement in that improved problem solving skills mediated the positive association between playing SVGs and academic performance [25]. Moreover, playing commercial VGs enhanced graduate skills (e.g. problem solving skills, communication) in university students, suggesting the potential efficacy of VG-based learning [58]. However, gaming habits (e.g. frequency and time of video gaming, genres of VGs) was found to have no influence academic skills in high school students [59]. The inconsistency between findings seemed to be the different use of measurement for problem solving skills. While self-reports were used to measure problem solving skills in [25] and [58], the measurement of academic skills (e.g. mathematics, science) was used in [59]. Although the longitudinal design of [25] suggested the potential positive influence of strategic VGs on problem solving skill, further studies that investigate the extent to which problem solving skills can be enhanced through VG playing and examine changes in relevant brain regions should be conducted.

The last cognitive function that is identified to be positively associated with VG is second language (L2) learning in that not only serious games but also commercial VGs provide players with the opportunity for language practice and acquisition [60]. Among various genres, MMORPGs, which are full with the opportunity of interaction between players, and between players and the VG environment in target language [61], are suggested as the efficient method for speaking practice [62] and are reported to facilitate the learning of L2 [63]. Players engaging in frequent interaction in VG environments were found to show strengthened functional connectivity (FC) within brain regions involved in language processing (i.e. left anterior insular/frontal operculum and visual word form area) [63]. Moreover, the attentional bias toward information relevant to the task was identified as the possible mechanism for facilitated L2 learning in MMORPGs in that the activation in DLPFC, parahippocampal gyrus and thalamus was higher in players of MMORPGs in the response to VG-related cues compared to neutral cues [63]. That is, playing MMORPGs are suggested to support L2 learning.

Although not only AVG but also other genres were found to be associated with cognitive enhancement, the transfer effect of VG experience was limited to specific underlying cognitive demands that was trained through VG playing [19, 20, 32]. Among various VG genres, AVGs, which activated multiple cognitive functions (e.g. attention, WM, hand–eye coordination) by providing players with physically and mentally demanding environments [46], showed the most varied effect of transfer [32]. However, unlike the suggestion that AVGs seem to improve the skill to infer regularities of presented information in the environment instead of the improvement of specific skill [64], AVG playing was found to require various information processing skill at lower level such as visual perception, attention skills and change detection [20]. AVG players, who efficiently tracked multiple moving objects compared to players of other VGs, were better at tracking multiple static objects [32]. FPS experience was also associated with the improvement in WM capacity but not with the improved inhibitory control [43]. That is, AVG experience was associated with the activation of specific brain regions [65]. LoL playing experience was associated with the activation in the frontal lobe compared to the activity with lower working loads (e.g. movie watching, SG experience) [66]. Moreover, AVGs did not show the transfer to different modality (i.e. auditory detection) and only players of AVGs that require faster attentional switch showed faster recovery from attentional blink [67]. Furthermore, the improvement of complex verbal WM was found in not memory matrix VG but AVG and match-3 VG that require strategic planning [32]. These findings suggested that VG playing did not show far transfer (i.e. general improvement in cognitive function to learn new skills) [20], supporting the common demand hypothesis that the VG-associated cognitive enhancement showed near transfer [20].

Taken together, six cognitive functions were identified to be positively associated with VG playing despite some discrepancies between findings (see Table 3). Different genres of VGs was associated with different aspects of cognitive function [5]. While AVG training was associated with attentional improvement, the training of match-3 VG resulted in better spatial WM [32]. Although the weaker association between FPS experience and cognitive enhancement in the sample including players with less FPS experience [68] questioned the positive association between VG playing and cognitive function, the reduction of VG playing significantly decreased not only self-reported gaming skills but also brain activities [69]. That is, as certain amount of VG experience is required to show cognitive enhancement (Anguera et al. 2015), VG experience was suggested to have the potential to enhance cognitive function and to show near transfer effect. However, most reviewed research articles were cross-sectional and did not examine the persistency of cognitive enhancement associated with VG playing. Although one study [11] examined the persistency of VG-associated cognitive enhancement by following up 9 months, VGs used in this study was not commercial VGs. That is, the extent to which not only AVGs but also other genres of VGs showed the transfer and the extent to which cognitive enhancement through commercial VG playing was persistent have been less examined. Thus, more studies, investigating not only the extent of transfer but also the persistency of cognitive advantage associated with VG playing, should be conducted in order to deepen the understanding of transfer effect of VG experience.

**Table 3:** The summary of research papers presented to describe the association between VG playing and cognitive function

Studies	Study design, participants	Measures	Associated cognitive functions	Additional information of results
[26]	Cross-sectional, 88 university students (N_Female_ = 50) (1) Console/computer VG players (N = 34) (2) Mobile VG players (N = 25) (3) Non-VG players	Cognitive tests (e.g. Go-No-Go task, N-back task)	Attention, WM	No enhancement in inhibition Similar enhancement in cognitive function between players using different gaming platforms
[19]	Intervention (15 h of training), young adults (1) WM-REAS 1 (N = 43, N_Female_ = 12) (2) WM-REAS 2 (N = 40, N_Female_ = 11) (3) AC (N = 44, N_Female_ = 12) (4) NC (N = 43, N_Female_ = 12)	Cognitive tests (e.g. Logical memory task, N-back task, Task switching) Behavior rating inventory of executive function	Attention	No improvement in WM capacity and fluid intelligence
[70]	Intervention (10 h of FPS training), university students without gaming experience (1) FPS group (N = 16) (2) Non-AVG control group (N = 6)	EEG recording during the attention visual field task that was completed before and after the training intervention	Attention	The modification of neural activity supporting spatial selective attention through FPS training Substantial improvement of selective attention in high-performing players in FPS group
[30]	Cross-sectional and intervention (1 h of AVG training), 29 males (1) LoL experts who were recognized as skilled-players with more than 2 years of AVG experience (N = 15) (2) Non-experts	EEG recording during the useful field of vision task that was completed before and after the AVG training	Attention	Better selective attention, attentional control and top-down modulated attentional allocation in LoL experts than in non-experts before the training Improved selective attention and attentional control in non-experts after AVG training No enhancement in accuracy in both groups
[31]	Cross-sectional, 119 participants (1) Players who reported to engage in AVGs or adventure games for more than 4 h weekly (2) Non-VG players	Attentional blink tasks with global orientation and local orientation	Attention	Faster recovery from attentional blink in VG players than in non-VG players when both global and local elements were attended
[32]	Intervention, 75 university students whose initial weekly gaming duration was less than 1 h (N_Female_ = 47) (1) Hidden-object VG group (N = 15) (2) Memory matrix VG group (N = 14) (3) Match-3 VG group (N = 14) (4) AVG group (N = 16) SG group	Cognitive tasks (e.g. attentional blink task, visual search/spatial memory task, complex span task)	Attention, WM	Enhanced recovery from attentional blink, improved skill to track multiple objects and better cognitive control to filter irrelevant stimuli out in AVG group Enhanced visual search in hidden-object VG group, memory matrix VG group and match-3 VG group Improved spatial WM in hidden-object VG group and memory matrix VG group Improved complex verbal WM in match-3 VG group and AVG group
[34]	Cross-sectional, 26 male young adults (age range = 18–26 years) (1) AVG players whose weekly playing time was more than 5 h (N = 12) (2) Non-VG players including players of non-AVGs	fMRI scanning during attention task	Attention	Reduced activation of MT/MST in AVG players in response to moving distracters Decreased recruitment of the fronto-parietal network (e.g. bilateral superior and middle occipital gyri, left inferior temporal gyrus), in AVG players compared to non-VG players
[33]	Cross-sectional, 131 participants (age range = 7–22 years) (1) AVG players (N = 56) (2) Non-VG players including players of non-AVGs	Attentional network test	Attention	Improved allocation of attention without speed-accuracy trade-off in AVG players compared to non-VG players
[36]	Cross-sectional, 58 male adults (1) AVG experts who were recognized as regional or national champions with more than 6 years of AVG experience (N = 28) (2) Amateur players of AVGs	MRI scanning	Attention, visuo-spatial function	Increased global and local plasticity of white matter network in the prefrontal network, the sensori-motor network and the limbic system in AVG experts compared to amateur players
[38]	Cross-sectional, 40 adults who were older than 60 years (1) VG players (N = 20) (2) Non-VG players	fMRI scanning during attentional network task	Attention	Better task performance in VG players than in non-VG players Higher activation in fronto-parietal regions (i.e. right DLPFC, left supramarginal gyrus, right angular gyrus, right precuneus, left paracentral lobule), right inferior temporal gyrus and left lingual gyrus in VG players than in non-VG players Higher FC between left paracentral lobule and right hippocampus, between left supramarginal gyrus and right DLPFC but lower FC between right precuneus and angular gyrus in VG players than in non-VG players
[28]	Intervention (30 h of training), 34 male university students (1) AVG training group (N = 17) (2) Control group playing SG	visual WM tasks (e.g. change detection task) and complex span WM task	WM	Improved accuracy of visual WM in AVG training group compared to SG training group No enhancement of complex span VWM in AVG training group
[42]	Cross-sectional, Experiment 1: (1) 48 AVG players whose weekly playing time was more than 5 h (N_Female_ = 5) (2) 49 non-VG players including AVG players whose weekly playing time was less than 1 h (N_Female_ = 6) Experiment 2: 47 participants from Experiment 1 (1) 24 AVG players (N_Femlae_ = 2) (2) Non-VG players (N_Femlae_ = 1)	Change detection task with more focus on the accuracy in both experiments	WM	Improved WM in AVG players compared to non-VG players regardless of the encoding time and better task performance in encoding more items Improved visual WM in AVG players compared to non-VG players regardless of the complexity of visual stimuli that were required to encode
[43]	Cross-sectional, 52 young adults (N_Female_ = 4) (1) FPS players (N = 26) (2) Non-VG players	Cognitive tasks (e.g. stop-signal task, N-back task)	WM	Better skill to monitor and update information that is relevant with task in FPS players than in non-VG players Comparable inhibitory control in both groups
[44]	Cross-sectional, 44 male adults (1) AVG players whose weekly gaming time was more than 3 and 4 h (N = 24) (2) Non-VG players including players of other VG genres	Multiple identity tracking task, color wheel task, useful field of view task	WM	Better skill to track multiple stimuli and to have precise representation of the stimuli (i.e. color, location) in AVG players than in non-VG players
[45]	Cross-sectional (1) Expert VG players (N = 11) (2) Non-VG players (N = 10) and Intervention (21.5 h of training), 82 adults whose weekly playing time was less than 1 h (1) AVG training group (N = 20) (2) SVG training group (N = 23) (3) TG training group (N = 20) (4) Control group	Visual and attentional tasks (e.g. multiple object tracking, VSTM), spatial processing and sptial memory tasks (e.g. mental rotation), executive control and reasoning tasks (e.g. task switching, tower of London)	Attention, WM	Better attentional function, WM, task switching and spatial processing in expert VG players than in non-VG players Comparable performance between training groups and control group
[46]	Cross-sectional (1) AVG experts with more than 4 years of AVG experience (N = 23) (2) Amateur AVG players whose AVG experience was less than 1 year (N = 22)	Cognitive tests (i.e. N-back task, spatial memory task) and resting-state fMRI scanning	Attention, WM	Improvement in global and nodal plasticity in salience network (e.g. ACC) and central executive network (e.g. DLPFC) in AVG experts than in amateur AVG players Enhanced FC between these networks in AVG experts compared to amateur AVG players
[48]	Correlational, 62 male participants with varied VG experience (mean age = 28 years)	Resting-state MRI scanning	Visuo-spatial function	The positive association of VG experience with GM volume in bilateral entorhinal cortex and left occipital lobe/interior parietal lobe Positive influence of logic/puzzle VGs on entorhinal GM volume but negative influence of AVGs on the GM volume in entorhinal cortex
[51]	Cross-sectional, 59 young adults (N_Female_ = 13) (1) AVG players whose weekly playing time was more than 6 h (N = 26, N_Female_ = 4) (2) Non-VG players	Cognitive tasks (i.e. virtual maze task, visual attention task)	Visuo-spatial function	The usage of response strategy in 80% of AVG players during the navigation of a virtual maze
[52]	Intervention (2-month of training), 48 young adults (1) AVG training group (N = 23, N_Female_ = 17) (2) Control group (N_Female_ = 17)	MRI scanning, cognitive tests (e.g. tunnel task) before and after the training	Visuo-spatial function	Increased GM volume in right hippocampus, right DLPFC, bilateral cerebellum in AVG training group compared to control group The association of increased volume in hippocampus with the adoption of allocentric orientation strategy
[53]	Cross-sectional, 62 male adults (1) RTS players whose weekly playing time was more than 6 h (N = 31) (2) Non-VG players who played RTS for less than 6 h weekly with less than 8 h of weekly VG experience	Operation span task, MRI scanning	Visuo-spatial function	Enhanced connectivity within occipital (e.g. left middle occipital gyrus) and parietal regions (e.g. superior parietal lobule) in RTS players compared to non-VG players Enhanced local efficiency in these regions in RTS players The positive association of RTS experience with alterations in the networks
[54]	Correlational, 152 adolescents who aged 14 years (N_Female_ = 80)	MRI scanning	Visuo-spatial function	The positive association of VG playing duration with cortical thickness in left DLPFC and left FEF when possible confounders (e.g. sex, age, scanner, socioeconomic status) were controlled
[55]	Cross-sectional (1) VG Players who played SVG or AVG for more than 15 h weekly (N = 17, N_Female_ = 4) (2) Non-VG players whose weekly playing time was less than 4 h (N = 17, N_Female_ = 4)	Weather prediction task, MRI scanning	Probabilistic learning	Better performance of categorization under the uncertainty in VG players than in non-VG players Highly activated brain regions (i.e. hippocampus, parahippocampal gyrus, precuneus, thalamus, attention-based areas (e.g. occipital visual areas), frontal gyrus and anterior cingulate gyrus) in VG players than in non-VG players
[56]	(1) Cross-sectional and intervention (50 h of training), AVG players (2) Non-VG players (2.1) AVG training group (2.2) Control group	Visual motion direction discrimination task, auditory tone location discrimination task	Probabilistic learning	More efficient use of both visual and auditory information in AVG players than in non-VG players Enhanced probabilistic inference in AVG training group
[57]	Intervention (8 h of training), 77 university students (age range = 18–22 years, N_Female_ = 44) (1) Puzzle VG training group (N = 42) (2) Cognitive training program group	Cognitive tasks for problem solving skills, spatial skills and conscientiousness (e.g. Insight test, mental rotation test, picture comparison task)	Problem solving skills	Improved task performance after the training only in puzzle VG training group Better performance in problem solving skills, spatial skills and conscientiousness in puzzle VG training group than in cognitive training program group
[25]	4-year longitudinal, 1492 adolescents who took part in at least 2 surveys over the four waves (mean age = 13 years and 10 months at the beginning of the study, N_Female_ = 758)	Self reports of problem solving skills, academic grades	Problem solving skills	Longitudinal relationship of strategic VG playing with self-reported skills to solve problems Indirect association of strategic VG playing with better academic performance through the improved problem solving skills
[58]	Intervention (14 h of training), 72 university students (1) VG training group (N = 36) (2) Control group	Self-reports for graduate skills including adaptability, communication skill, resourcefulness (e.g. communicative adaptability scale)	Problem solving skills	Enhanced communication skills, adaptability and resourcefulness in VG training group compared to control group
[59]	Correlational, 479 adolescents in high schools (N_Female_ = 209)	Problem solving test, self-reported academic performance	Problem solving skills	No association between VG playing behaviors (e.g. VG genres) and problem solving skills despite small significant association of higher problem solving skills with the frequency of console gaming and the experience of computer gaming No association of academic performance with VG genres and self-reported gaming skills
[62]	Intervention, 8 university students	Virtual ethnography tracking the process of learning, tutorial exams	L2 learning	More active usage of English Increased patience in reading Higher level of motivation for the communication with other players

Notes. WM-REAS 1 stands for WM-reasoning group, WM-REAS 2 stands for adaptive WM-reasoning group, AC stands for active control group, NC stands for no-contact/passive control group, EEG stands for electroencephalography, (f)MRI stands for (functional) magnetic resonance imaging, VSTM stands for visual short-term memory, ACC stands for anterior cingulate cortex

## Modulating factors

The positive association between VG playing and cognitive function has been demonstrated. However, there is individual difference in the extent to which players show cognitive enhancement [70]. It is because some factors influence the plasticity and individual responses to the VG training [20]. Thus, this section reviews five factors that are identified to modulate the association between VG playing and cognitive enhancement through the review of searched literatures.

The first modulating factor is VG expertise. VG expertise influences cognitive processes that players adopted during VG play. While novice players are more likely to use top-down process where attentional resources were allocated through the strategic control of gaming behavior, VG experts are more likely to use bottom-up processes where attention is automatically allocated to psychologically salient gaming cues as a result of rich experience [63]. Players with better VG expertise also prioritized skills to strategize during LoL playing compared to lower-ranking players who prioritized action skills [65]. That is, VG expertise influenced the activation of different cognitive processes during VG play. Moreover, as VG expertise was found to be closely associated with the difference in the baseline speed of visual attention [33], it seemed to influence attentional benefits associated with VG playing.

Secondly identified modulating factor for the individual difference is age in that different media were used for longer time in younger children [71]. After peaking at the age of 13 or 14 years, the time of VG playing decreased with age [23]. Age-related difference in VG playing time suggests that the effect of video gaming potentially has more influence on cognitive function enhancement in younger adults than older adults [72]. Age also influences the engagement in VG training. As the specific population considered in designing AVGs is young adults [5], older adults reported lower engagement in AVG training compared to the training of other genres of VGs [73]. Moreover, age is closely related to cognitive functions and the performance of the task in that the functional connectivity of brain develops with age [74]. While substantial neuro-plasticity was found in younger children [5], age-related decline in cognitive control was found [75]. It was also found that the task performance of younger children with relatively slow and less precise attention process became better when their performance was supported by the provision of temporal cues for attentional responses [33].

As age is associated with cognitive function, thirdly identified factor is baseline cognitive function (e.g. reasoning skill, attentional skill). Baseline cognitive function influences the choice of VG engagement in that cognitive ability was better in players, who chose regular AVG playing, than in individuals who barely played VGs [5]. It also influences the extent to which cognitive function would be enhanced through VG playing. Children with more attentional deficits were found to gain greater attentional enhancement through the computerized training and to show persistent effect in 9 months [11]. Players with lower baseline reasoning skills were also found to gain more cognitive benefits, such as better divided attention and faster perception of stimuli [19]. However, consistent with the influence of baseline GM in striatum on the degree of skill acquisition in learners [76], young adults with higher level of baseline modularity in brain showed higher cognitive benefits after VG training where WM and reasoning function was involved [77]. It is plausible that the modulating role of baseline cognitive function depends on the aspect of cognitive function trained in VGs. Although further studies should be conducted to examine this idea, baseline cognitive function are suggested to modulate the cognitive benefit of VG playing by influencing the choice of VG genre and the extent of cognitive enhancement.

Cognitive function that was identified as one modulating factor was associated with gender in that males were better at inhibiting distracters and sustaining attention [26]. That is, fourthly identified factor is gender that influences gaming habits that were reported to be associated with the extent of enhancement and types of cognitive functions improved [26]. Although both female and male AVG players gained similar attentional advantage despite the asymmetric gender distribution in the frequency of gaming [33], gender influences gaming time and styles. While Huang et al. [26] found males more frequently engaged in VGs than females, Dindar [58] reported that females played VGs more frequently than males. Despite the discrepancy in the frequency of gaming between males and females, it was confirmed that the duration for VG play was longer in males than females [23, 59, 78] and that males preferred to play AVGs [26]. The increased playing time in males was associated with the genre of VGs (e.g. whether it is played by multi-players) [79]. Moreover, males chose computer as the gaming platform compared to females [26]. Although the difference in cognitive enhancement between gaming platforms (i.e. mobile and console) was not significant [26], the choice of gaming platform was found to be associated with the motivation (e.g. social interaction) for VG engagement. While males prefer to play VGs focusing on competition (e.g. AVGs or SGs), females like to play TGs [23]. Taken together, gender, which is associated with the engagement habits in VGs, indirectly modulates the association between VG playing and cognitive function enhancement.

Based on the gender difference in the choice of gaming platform, the last factor that is identified to modulate the association between VG playing and cognitive functions is motivation. Individuals with higher level of motivation engaged in trainings more voluntarily, performed better in trainings and showed improved WM than those with lower level of motivation [80]. Players, who were more motivated to communicate in VGs through the experience of social rewards (e.g. positive expressions) in gaming environments, also showed attentional bias to communication-relevant stimuli that was associated with promoted L2 learning [63]. Moreover, the existence of motivational factor during the VG playing appears to influence the functional changes of the brain associated with the training [81]. Players, who experienced more fun and relatively less frustration during VG playing with better performance, were more motivated to engage in VGs and showed more functional changes in the relevant brain regions [81]. However, when monetary reward was given for game playing, motivation was not found to significantly influence the effectiveness of VG training [19]. Taken together, although monetary reward minimized the role of motivation in gaming engagement, motivational factors were suggested to be considered in investigating the association of VG playing with behavioral and neural changes [63].

As five factors, identified to influence the individual difference in the association between VG playing and cognitive improvement, interact each other in the modulation of the association, it is difficult to conclude which factor exerts more influence on the individual difference in the association between playing of VGs and cognitive enhancement. Moreover, there are other factors that are not introduced in this section. For example, the personalities of players seem to influence the motivation for VG playing [9] and the expression of motivation during the VG play [82]. Personality traits can also influence learning effects in that introverted players can get more benefits of language learning from playing VGs where they simulate the practice more freely compared to the traditional learning [61]. Moreover, players showed the difference in the results of learning depending on their learning styles [24]. As the individual difference in VG gains seemed to be explained by not only the duration of VG paying but also the variation in learning trajectories [83], other factors excluded in this review should be considered and further studies, including all these factors, should be conducted in order to understand what modulates the association between VG playing and cognitive enhancement.

## Conclusions

Unlike the emphasized negativity of VG playing, VGs are suggested to enhance cognitive functions. As VG playing has become one aspect of life in young people [84], it is important to understand the association between video gaming and cognitive function in a more balanced view toward VG playing. Thus, this paper discusses genres of commercial VGs, cognitive functions that are identified to be positively associated with VG playing and modulating factors. It is found that different genres of VGs are associated with different aspects of cognitive functions, that AVGs are identified as the VG genre resulting in most varied transfer, and that factors (e.g. age, gender) influence the association of VG playing with cognitive function. Moreover, despite the concern about the usage of VGs or computerized programs as the primary intervention for the improvement in brain function [19], VGs, demonstrating the association with structural changes in brain regions, have the potential to be used as an intervention program for patients showing decreased volume in brain regions such as hippocampus [36, 52].

Although this review contributes to the understanding of commercial video gaming and its potential effect, the findings of the review should be interpreted by considering three identified research gaps. One identified research gap is the limited generalizability resulting from the absence of standardized definition for VG players. More studies have been conducted by focusing on AVGs compared to other genres of VGs and the criteria for the status of VG players were different between studies. While non-VG players were mostly defined as individual with less or no VG experience, non-AVG players were classified as non-VG players in some studies (e.g. [33], [34]). The different classification criteria seems to underestimate the potentially influence of other genres of VGs on cognitive functions and makes it difficult the comparison between studies difficult. The other identified gap is habits of VG playing were usually based on self-reports. As self-report measure is based on autobiographical memory, it could result in inaccurate report of their frequent behaviors [23]. In order to understand the link between VG playing and cognitive function, further studies, including more reliable measure for VG playing habits, should be conducted. Another identified gap is the scope of cognitive function that has been investigated in relation to VG playing. While more studies focused on attention, studies for higher cognitive functions have been less conducted. Although the enhancement in inhibition was not associated with frequent VG playing [26], cognitive control was positively associated with AVG experience [36]. The transfer into complex verbal WM in AVG and match-3 game training groups also suggested the potential of VG training in the enhancement of higher order executive processes [32]. As the change in some cognitive functions is slow [19], it is plausible that higher cognitive function requires more playing time for the change. In order to resolve the discrepancy between findings and to deepen the understanding of the association between VG and higher cognitive function, mores studies, investigating more various aspects of cognitive function, should be conducted. Therefore, more studies, considering these research gaps, should be conducted in future in order to deepen the understanding of the influence of VG playing on cognitive function.

## Data Availability

Not applicable.

## Abbreviations

VG(s): Video game(s)
TBI: Traumatic brain injuries
SPD: Sensory processing dysfunction
ADHD: Attention deficit/hyperactivity dysfunction
TDC: Typically developing children
TGs: Traditional games
SGs: Simulation games
SVGs: Strategy video games
AVGs: Action video games
FGs: Fantasy games
RPGs: Role-playing games
MMORPGs: Massive multi-player online RPGs
RTS: Real-time strategy
TBS: Turn-based strategy
FPS: First-person shooters
TPG: Third-person games
LoL: League of Legends
WM: Working memory
PFC: Prefrontal cortex
DLPFC: Dorsolateral PFC
GM: Gray matter
L2: Second language
